# The role of individual behavioral traits on fishway passage attempt behavior

**DOI:** 10.1002/ece3.7964

**Published:** 2021-08-01

**Authors:** Angus J. Lothian, Martyn C. Lucas

**Affiliations:** ^1^ Department of Biosciences Durham University Durham UK

**Keywords:** behavioral traits, biotelemetry, boldness, fish passage, *Salmo trutta*, wildlife pass

## Abstract

Variations in behavioral traits are widely recognized to drive animal behaviors exhibited within a population. However, information on how behavior traits influence behavior in anthropogenically modified habitats is lacking. Many habitats have become highly fragmented as a result of human processes. To mitigate this and improve habitat connectivity, wildlife passes are increasingly employed, with the aim of enabling animals to move freely between habitats. However, wildlife passes (e.g., fishways) are not always effective in achieving passage and it remains uncertain what factors play a role in an individual's likelihood of passing successfully. This study measured three behavioral traits (boldness, exploration, and activity) in juvenile brown trout (*Salmo trutta*; *n* = 78) under field conditions within a river and tested whether these behavior traits influenced both the passage success and the behaviors exhibited during upstream fishway passage attempts. Although behavioral traits were found and collapsed into two behavioral trait dimensions, behavioral traits had low repeatability and so did not contribute to a personality spectrum. Boldness was found to negatively influence the number of passage attempts carried out by an individual and to positively influence passage success, with bolder individuals carrying out fewer attempts and having an increased probability of passage success. No behavioral traits were found to be related to other passage metrics (passage success, Time until First Attempt, and Passage Duration) during the first passage. But all three behavioral traits were significantly negatively related to the changes in passage behaviors at consecutive, successful passage attempts, with bolder, more exploratory and more active individuals passing through a fishway quicker on the second passage than on the first. This study suggests that bolder and more active individuals may perform better during fishway passage attempts, particularly within rivers where multiple barriers to movement exist.

## Graphical abstract

Information on how behavior traits influence behavior in anthropogenically modified habitats is lacking. We show that an individual's boldness influences the interactions fish have with fishways (wildlife passes) and that boldness, exploration, and activity influence interactions at multiple fishways. These interactions may lead to fitness consequences and may result in selection for specific behavioral and physiological characteristics.

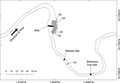

## 1 INTRODUCTION

The field of animal personality (also referred to as behavioral syndromes, temperament, and coping strategies; Dingemanse & Réale, 2005) is a rapidly expanding area of research that attempts to partition animal behaviors into consistent and repeatable traits across time and across environmental stimuli (Dingemanse et al., 2010; Dingemanse & Réale, 2005; Hertel et al., 2020; Sih & Bell, 2008; Sih, Bell, Johnson, & Ziemba, 2004; Sih et al., 2012). Commonly, these traits have been placed on easy‐to‐interpret behavioral axes or continuums to define behavioral traits (or behavioral types or behavioral tendencies; Dingemanse & Réale, 2005; Stamps & Groothuis, 2010), such as boldness (Álvarez & Bell, 2007; Brown et al., 2005), activity (Montiglio et al., 2010; Smith & Doupnik, 2005), sociality (Cote et al., 2011; Hirsch et al., 2017), and aggression (Bell & Sih, 2007; Duckworth, 2006). Correlations between repeatable behavioral traits then make up and constitute personality traits (Bell, 2007; Sih & Bell, 2008).

The role of behavioral traits in influencing how individuals behave has been extensively hypothesized and investigated over the last half‐century (Conrad et al., 2011; Dingemanse et al., 2010; Sih, Bell, & Johnson, 2004; Sih et al., 2012). For example, the boldness trait has been shown to influence dispersal distance, migration propensity, food acquisition, and adaptation to novel environments (Chapman et al., 2011; Cote et al., 2011; Fraser et al., 2001; Thorlacius et al., 2015; Wilson et al., 1993). However, little work has been carried out to further our understanding of how individual behavioral traits are related to behaviors exhibited when interacting with anthropogenic changes to the natural environment (Sih et al., 2011, 2012).

Habitat fragmentation occurs largely as a result of anthropogenic processes, affecting both terrestrial (e.g., road construction, deforestation, agriculture) and aquatic (e.g., construction of dams, weirs, and culverts) ecosystems (Haddad et al., 2015; Jones et al., 2019). This constrains the natural movement of animals, the consequences of which range from suboptimal resource acquisition (Andren, 1994; Saunders et al., 1991) to mortality from interactions with human‐built structures (Brackley et al., 2018; Haigh et al., 2014; Thorstad et al., 2008). These negative consequences may be either direct (e.g., collisions with motor vehicles during road crossings, strikes from hydropower turbines) or indirect (e.g., excessive energy expenditure, increased predation). In order to mitigate against habitat fragmentation and the associated consequences, wildlife passes are increasingly constructed at barriers to provide safer access routes between habitat patches for target species. Such wildlife passes include road underpasses and overpasses (Beben, 2016) and fish passes [=fishways] (Noonan et al., 2012; Silva et al., 2018). Despite this, wildlife passes do not always mitigate the effects of barriers, with differential passage success (defined as the proportion of animals attempting to move across an obstacle that ultimately succeed in passing the obstacle) being reported within and between species and pass designs (Woltz et al., 2008; Noonan et al., 2012).

Variability in passage success has been attributed to several biological factors, for example size of animal, sex, and proximity to the breeding period (Bunt et al., 2012; Noonan et al., 2012). However, understanding an animal's use of a wildlife pass is not simple. Where the passage success is not 100%, it is unclear whether this is a result of the wildlife pass not functioning properly (i.e., not encompassing the performance attributes of the target species), or whether the animal itself lacks the motivation, or has a behavioral predisposition to not use it (Castro‐Santos & Haro, 2010). This is particularly important to distinguish in riverine environments where, due to the linear nature of rivers, aquatic fauna are limited to bidirectional movements and often cannot use another route around an obstacle.

Recent theoretical work on fish passage has postulated that bolder individuals may have an increased chance of succeeding in upstream passage via a fishway at a dam (Hirsch et al., 2017). This theory is driven by the idea that bolder individuals are more likely to move to empty areas sooner than shy individuals, and thereby increase the speed and distance of dispersal within a population (Chapple et al., 2012; Fraser et al., 2001). If this were to be the case, where bold individuals are more likely to succeed in passage of dams and weirs, then bold fish might gain an evolutionary advantage over shy fish, particularly if habitat upstream of the dam increases overall fitness. Recent laboratory experiments in an artificial flume‐cascade simulating barrier passage appear to support this hypothesis, showing that boldness is positively related to upstream passage rate for brown trout (*Salmo trutta*; Jones et al., 2021). However, Landsman et al. (2017) failed to identify any relationship between an individual's boldness and the probability of passage success at a nature‐like bypass for rainbow smelt (*Osmerus mordax*) in an *in situ* study.

The costs of barriers to movement on an individual's fitness are, however, not solely dependent on whether the animal is able to pass the barrier or not, but also dependent on the behaviors exhibited while doing so (Castro‐Santos et al., 2009; Silva et al., 2018). The array of behaviors exhibited by individuals during passage of barriers to movement, such as the number of passage attempts carried out, the speed and duration of approach to the barrier, the search for passage routes, and the speed of passing the barrier, can all impact on an individual's fitness. These impacts may occur either through increased energy expenditure (although this may only account for a small fraction of available energy), or through increased exposure to predation (Silva et al., 2018). For juvenile fish dispersing upstream or that have been displaced downstream as a result of elevated flows, failure to pass upstream or depletion of motivation as a result of several passage attempts, or increased passage duration, may ultimately prevent them from accessing areas of underutilized and thusly more abundant resources, impacting on their long‐term fitness with potential diminished growth opportunity (Forty et al., 2016). As passage behaviors observed at fishways are not the same across all individuals (e.g., some involve few attempts and pass quickly, others involve more attempts and may pass more slowly), it is important to understand the drivers of these variations. Environmental variables (e.g., river flow) are recognized to influence passage behavior (Dodd et al., 2018; Winter et al., 2016), but information on the relationships between behavioral traits and passage behavior is missing.

The keys elements of fish passage at a barrier with a fishway are as follows: the approach to the obstacle, the search for routes to pass it (often associated with one or more attempts) and the passage of the obstacle. These behaviors can be expected to be associated with boldness (approach of obstacle and entering passage route), exploration (searching for passage route), and activity (searching for passage route and passing the weir). For this reason, this study aimed to measure these three behavioral traits from juvenile brown trout in an open field experiment, and investigate the role of these in determining individual differences in passage behaviors (time taken to approach the fishway, time taken to pass through the fishway, and the number of passage attempts made) and the ultimate success in passing the weir using a fishway.

In northern temperate zones, river fragmentation has heavily impacted on brown trout populations. This is largely a result of size‐related passage ability and selectivity by barriers to migration whereby larger individuals are more likely to succeed in passing upstream of an obstacle (Jones et al., 2021; Lothian et al., 2020; Noonan et al., 2012). Given that the reproductive component of a population can stem from any of the phenotypes which can be classified into discrete size groupings (Birnie‐Gauvin et al., 2019; Ferguson et al., 2019), smaller phenotypes can be severely curtailed in their migratory range, potentially being denied access to suitable spawning grounds. Juvenile brown trout can also exhibit high degrees of dispersal, both upstream and downstream (Ferguson et al., 2019), and so are impacted by barriers to movement. Although there are differences in the expression of behavioral traits in juvenile and adult individuals (Sih & Bell, 2008), juvenile brown trout were selected for this study as they are more easily accessible year‐round, and they generally lack certain intrinsic factors (such as sexual maturation) that might alter behavioral traits and passage motivations.

The predictions for this study were that those individuals which were bolder, more exploratory and more active would (a) approach the fishway quicker, (b) exhibit fewer passage attempts before completing passage, (c) have a shorter passage duration, and (d) have a greater passage success than those which were shyer, less exploratory, and less active. Furthermore, behavioral traits were compared against the passage behaviors observed during multiple passages to investigate the role that behavioral traits have on the cumulative impact of multiple passage.

## 2 MATERIALS AND METHODS

### 2.1 Study site

**FIGURE 1:**
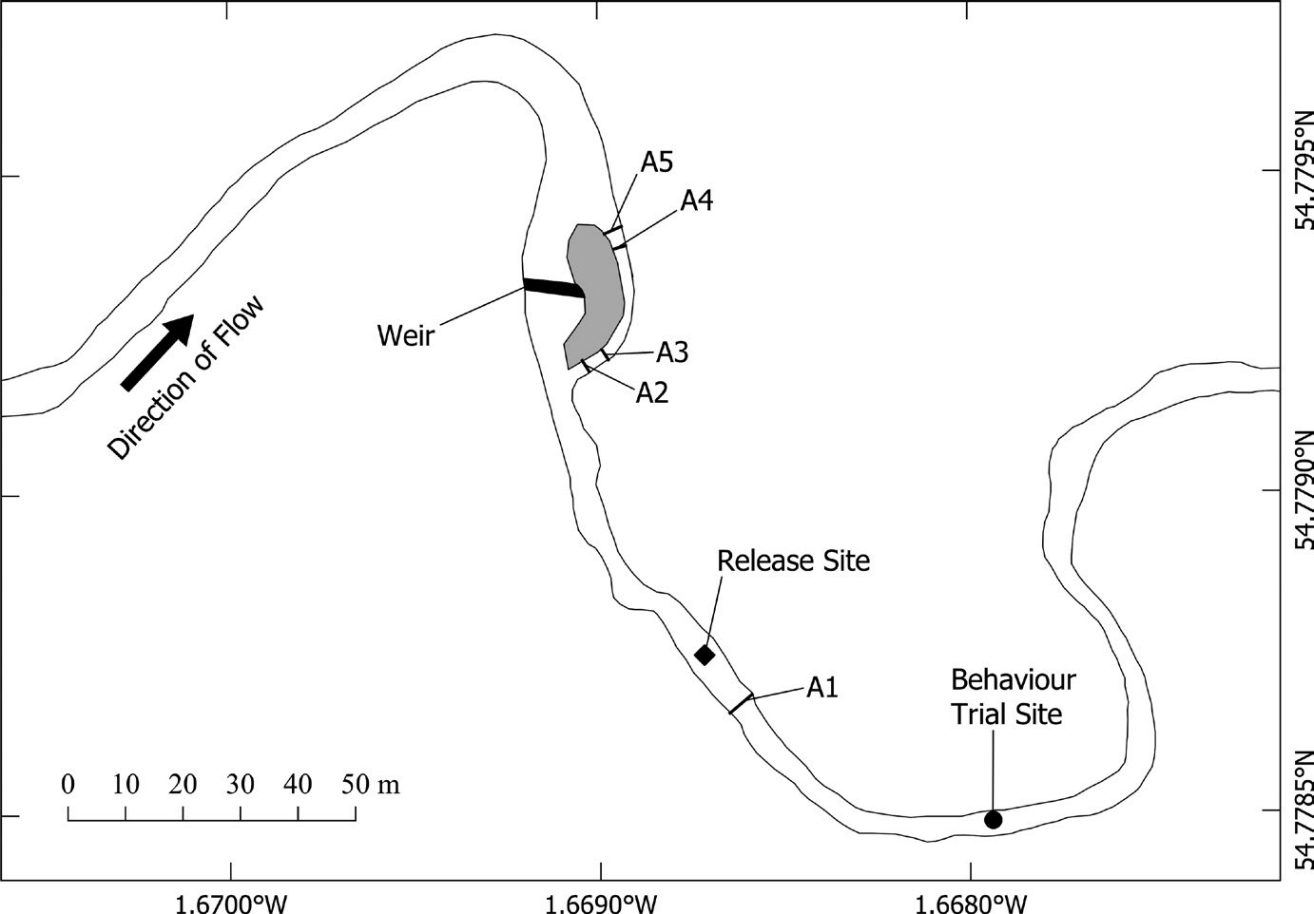
Map of the study site on the River Deerness, NE England, with locations of PIT antennas (A1–A5) and the sites where behavior trials of brown trout were carried out (filled dot) and where fish were released (diamond)

This study was conducted on the River Deerness, northeast England, between 20 July and 14 September 2018. The Deerness is approximately 19 km in length, drains an area of 36 km^2^, and in its lower reaches has an average flow of 0.5 m^3^/s (Winter et al., 2016). A vertical ~2‐m high weir (54°46′45.53″N, 1°40′8.80″W; Figure 1) was determined to be a barrier to fish movement, and a nature‐like bypass was installed in 2013. A nature‐like bypass is designed to mimic the river conditions as best as possible (Jungwirth et al., 1998). This bypass is 36 m in length and 2 m wide, with a slope of 2.7% (Tummers et al., 2016). The vertical weir currently acts as a dam with water only overflowing it during high‐flow events, therefore almost entirely diverting the river through the bypass channel. A telemetry study using wild brown trout translocated from home sites upstream to downstream of the weir, immediately after the construction of the bypass, showed that 70.2% of those translocated returned upstream (Tummers et al., 2016), suggesting that the fishway enables upstream passage, but that the barrier has not been fully mitigated, providing a possibility to examine differential passage success and behavior.

### 2.2 PIT logging station network

One half‐duplex (HDX) Passive Integrated Transponder (PIT) station, full‐stream width, single swim‐through antenna (A1), was constructed downstream of the release site (constructed 25 July 2018; one day after the first release group) to provide information on fish moving downstream from the release point (Figure 1). Two further PIT stations (constructed 20 July 2018), each consisting of two full‐channel width, swim‐through antennas, were built at the entrance (A2 and A3) and exit (A4 and A5) of the bypass. All antennas consisted of a single winding of 6 mm^2^, 777 strand, braided, oxygen free, copper wire encased in an insulating Polyvinyl Chloride (PVC) layer (FS Cables Ltd). Two antennas were constructed at both bypass PIT stations to ensure no fish were missed either entering or exiting the bypass (because of possible detection blocking by a tagged fish “sitting” adjacent to a single antenna; Cooke et al., 2012). Therefore, data from A2 and A3, and A4 and A5, were combined and treated as single entrance and exit antennas, but with a focus on the data obtained on A2 and A5. Each station was operated using one reader unit (in‐house built, Texas Instruments SX2000) via a tuning box that was placed on a wooden pole above flood height, and connected to the reader unit by buried, shielded twin‐ax cable. A1 was controlled by a single primary drive, whereas the two bypass stations were controlled by time‐synchronized primary (A2 and A4) and secondary (A3 and A5) drives. PIT stations were powered by two 12V 110Ah leisure batteries connected in parallel which were replaced every 3–4 days. Data (date, time, antenna number, PIT tag ID) stored on a compact flash drive housed within the reader unit were downloaded from PIT stations during each battery change.

Antenna functionality and range were checked manually on each visit; all readers and antennas were operational for 100% of the study period. Detection ranges were tested with a 23 mm PIT tag, and identified to be ~0.3 m either side of the antenna plane. No fish were identified as having been missed by any antenna.

### 2.3 Fish capture

For brown trout, the River Deerness is primarily a nursery stream and during summer almost all trout are juveniles. No Deerness trout have been stocked in living memory. From 2013 to 2015 length frequency distribution analysis of brown trout caught in the Deerness summer surveys showed four modal length groups, indicative of consecutive age groups (Tummers, 2016). Since partial connectivity restoration in the river (Tummers et al., 2016) this has adjusted to just show two modal groups reflecting age 0+ and 1+, indicating that almost all trout emigrate from the stream at or before an age of 2 (M. Lucas, personal observation) and very few remain to maturity. For this study, juvenile brown trout, identified by parr‐markings along their flanks, were captured by electrofishing 100–550 m upstream of the weir and translocated to downstream of the weir. Translocation of fish from upstream to downstream of the weir instigates homing in the brown trout, a response that has been well documented for brown trout in rivers with and without barriers (Armstrong & Herbert, 1997; Forty et al., 2016; Halvorsen & Stabell, 1990; Tummers et al., 2016), thereby providing each individual with a high motivation to use the bypass to return to their home pool. Electrofishing of three ~75 m sections was conducted on 12 days (one section per day, for the first 6 days, and then the lowest two sections for the last 6 days) between 24 July and 22 August 2018 using pulsed DC electrofishing. Between four and nine fish of suitable size (>12 cm long, age 1 or more) were captured for trials in each electrofishing session. Captured fish were transported ~300 m to the behavior trial site by handheld buckets filled with river water (Figure 1). Once at the tagging site, fish were placed into separate tanks (40 L) containing river water and which were also continuously aerated. Partial water changes with river water were carried out hourly to maintain water quality and temperature close to that of the river. Fish were given at least 1 hr to recover before commencement of trials.

### 2.4 Behavior trials

Many fish behavior and personality assays are conducted under laboratory conditions (Greenberg, 1992; Hirsch et al., 2017; Johnsson et al., 1996; van Leeuwen et al., 2016; Metcalfe et al., 2016). However, fish do not behave in the same manner under laboratory conditions in comparison with natural settings (Höjesjö et al., 2002; van Leeuwen et al., 2010). Therefore, this study adapted field behavioral assays (Brown et al., 2005; Landsman et al., 2017) and conducted the trials *in situ* in the river with a natural river flow and substrate. The behavior trial procedures (detailed in Sections 2.4.2, 2.4.4) were adapted from those used by Cote et al. (2011).

#### 2.4.1 Trial enclosure

**FIGURE 2:**
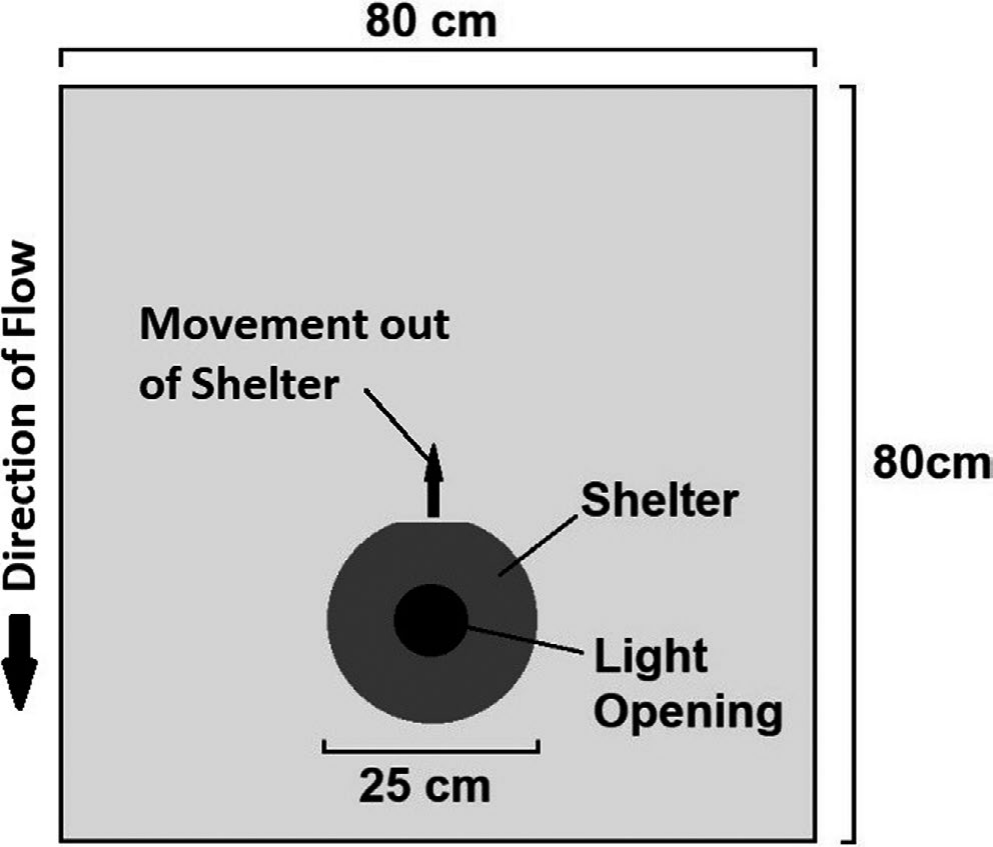
Plan view of behavior trial enclosure (not to scale)

The self‐supporting enclosure (80 × 80 × 40 cm; Figure 2) for behavioral typing trials was constructed from wire fencing of mesh size 13 × 13 mm and held in shape by a wooden frame around the top, above the water level. The enclosure was placed downstream of the release site in a shaded (>95% tree cover) section of shallow (~15 cm depth) river with slow, steady flow (~0.05 m/s) and a substrate consisting of small pebbles and sand. To allow river water to flow through the enclosure unhindered and to limit the effect of the enclosure frame on the hydrodynamics within the enclosure no vertical corner supports were present. An up‐turned pot with an opening cut out of one side was placed off‐center (Figure 2), with the opening directed upstream, to provide a shelter for the fish. Another up‐turned pot was placed over the shelter to prevent the fish from leaving the shelter during acclimatization, but which was then removed to start the behavior trials. Fish behavior was monitored using a camera (Finepix F600EXR, Fujifilm) positioned perpendicularly directly overhead with no human presence within 15 m of the enclosure for the duration of the trial.

In total, 78 fish were behaviorally typed (Table 1), ranging from four to nine fish per day, with trials carried out between 1000 and 1515 hr. Behavior trials were conducted between 24 July and 22 August 2018, but were not carried out on days where rain was forecasted, nor on days that were windy (>24 km/h wind speed for Durham City [~5 km from the trial site]; similar to the criteria used by Landsman et al. (2017)) to ensure that there was a) suitable water clarity for filming (rain and wind would disturb the surface layer and cause turbidity within the water column), and b) no disturbance to the fish that might alter their behavior.

**TABLE 1:** Summary of the fork length and mass of behaviorally assayed brown trout, and the number of fish that did and did not leave the experimental shelter during assays

Date	Left shelter	No. tagged	Fork length (mm)		Mass (g)	
			Mean (*SD*)	Range	Mean (*SD*)	Range
24/07/2018	Yes	4	153 (18)	132–176	41 (15)	27–62
	No	1	183 (n/a)	183–183	79 (n/a)	79–79
26/07/2018	Yes	5	141 (11)	126–155	33 (6)	24–40
	No	3	144 (22)	127–169	36 (18)	23–56
30/07/2018	Yes	6	141 (8)	131–154	32 (5)	25–40
	No	0	—	—	—	—
01/08/2018	Yes	6	136 (13)	122–158	29 (8)	21–44
	No	2	123 (4)	120–125	21 (1)	20–21
02/08/2018	Yes	2	146 (14)	136–156	35 (11)	27–43
	No	4	139 (11)	130–156	31 (9)	24–44
07/08/2018	Yes	3	140 (4)	138–145	31 (3)	28–33
	No	4	151 (15)	131–165	42 (12)	25–53
09/08/2018	Yes	5	146 (16)	126–164	34 (11)	21–48
	No	2	166 (42)	136–196	55 (37)	28–81
10/08/2018	Yes	3	165 (14)	155–182	54 (21)	39–78
	No	6	165 (15)	129–215	53 (31)	23–103
16/08/2018	Yes	4	148 (30)	124–191	40 (26)	22–79
	No	3	142 (9)	132–150	32 (7)	24–37
17/08/2018	Yes	6	153 (23)	136–197	42 (19)	28–78
	No	1	140 (n/a)	140–140	31 (n/a)	31–31
21/08/2018	Yes	3	148 (8)	141–156	38 (7)	33–46
	No	1	131 (n/a)	131–131	24 (n/a)	24–24
22/08/2018	Yes	2	135 (3.5)	132–137	27 (3)	25–29
	No	2	129 (3)	127–131	24 (3)	22–26
Total	Yes	49	146 (16)	122–197	36 (14)	21–79
	No	29	148 (24)	120–215	40 ( 22)	20–103

After the initial behavior trial period, a subset of fish (*n* = 12) were recaptured (4 and 5 September 2018) during electrofishing 0–550 m upstream of the weir and reassayed to measure repeatability of behavior scores. More than 12 fish were preferred for retriallng, but no more could be caught. The time between first and second behavioral assays ranged from 13 to 41 days for the 12 fish.

#### 2.4.2 Boldness trial—shelter departure latency

Boldness was measured as the latency time for a fish to leave the shelter into the natural environment of its own volition. A fish was placed inside the shelter and allowed to acclimatize for 20 min. After this time, the door to the shelter was removed, enabling the fish to travel freely from the shelter into the enclosure. The fish was given a maximum time period of 15 min to leave the shelter. For those fish that left the shelter within that time, a shelter departure latency (s) was calculated as the time taken to leave. For those fish that had not left the shelter, they were given a ceiling value of 900 s (however, due to them not leaving the shelter, other behavior scores could not be obtained and so these fish were removed from certain analyses). A fish was defined as leaving the shelter when its eyes passed the plane of the shelter opening for more than 10 s. This was determined post‐experiment through analyzing video footage.

#### 2.4.3 Exploration trial—area of enclosure explored

After the fish had left the shelter, its exploration of the enclosure was monitored for 10 min by video recording. Video recordings were analyzed following the methods used by Hirsch et al. (2017). Frames (image stills from the video) were extracted from the video footage at one second intervals using the ffmpeg software package (ffmpeg.zerano.com). One second intervals ensured sufficient time for fish displacement between successive images without losing detail in the movement which might have resulted in under‐representative area explored being calculated. It was assumed that the fish moved in a straight line for each 1 s interval. Each frame was then analyzed in ImageJ (imagej.nih.org). The *x*‐ and *y*‐coordinates of the midpoint between the eyes of each fish were extracted from each image. After extracting coordinates, plots of each fish's movement were made. For each plot, a 10 × 10 grid (equating to 8 × 8 cm grid squares) was overlaid onto the movement tracks, and the number of squares visited was counted and the area of enclosure explored (cm^2^) calculated, resulting in a value for exploration.

#### 2.4.4 Activity trial—time spent active

Activity, reported as the time (s) spent active during the exploration trial, was calculated as the number of frames in which a fish was displaced from the previous point of observation. Although activity and exploration are often heavily correlated (Cote et al., 2010, 2011), they were deemed separate measures of behavioral traits as a fish might continue being active within an area that it had already explored.

### 2.5 Fish tagging

After behavioral typing trials, fish were tagged with HDX PIT (23 × 3.4 mm, 0.6 g in air, Oregon RFID, Inc.) tags. This was done after the behavioral typing trial to ensure the effects of handling and tagging did not influence the results of the behavior trials (Wilson et al., 2017). The influence of tagging procedures and tag‐implantation on behavior has been heavily debated, but body mass and fork length tag burdens of <2% have been shown to not alter mortality or growth rates, nor individual behavior (Brown et al., 2011; Jaddot et al., 2005; Vollset et al., 2020). Mean body mass tag burden within this study was 1.8% ± 0.6% (*SD*; range = 0.6%–3%).

Fish were lightly anesthetized in a buffered solution of river water and tricaine methanesulphonate (MS‐222; 100 mg L^‐1^). Following sedation, each fish was measured in length (fork length; mm) and mass (g). A 3–4 mm incision was made anterior to the pelvic girdle on the ventral surface of the fish before a PIT tag was inserted into the body cavity. No sutures were used to close the incision. Tag retention in juvenile Atlantic salmon (*Salmo salar*) tagged with 23 mm PIT tags without suturing the incision has been shown to be high (97% retention; Larsen et al., 2013). All fish were left to recover in aerated tanks of river water until they were deemed to have completely recovered from the anesthetic (~1 hr). Recovered fish were then released into a slow moving glide and pool with overhead tree coverage and a complex of tree roots in the water ~60 m downstream of the weir. All procedures were conducted in accordance with United Kingdom Scientific Procedures Act 2003 under a Home Office issued license.

### 2.6 Environmental variables

Brown trout movement, including on the Deerness, can be influenced by river level (Winter et al., 2016) potentially through motivation, but also by modifying hydraulic conditions at obstacles and bypasses (Silva et al., 2018). Mean daily river discharge was obtained from the Environment Agency flow gauging weir at Burnhall on the River Browney (of which the Deerness is the major tributary) ~15 river km downstream of the study site. River stage at the study site and at Burnhall are highly positively correlated (ANOVA: *r* ^2^ = 0.82; data compared for time period 1 October 2014 to 31 May 2015).

### 2.7 Statistical approach

All analyses were carried out in RStudio (v1.2.1335) using R (v3.6.0; R Core Team, 2014). The frequency distributions of the behavior traits were assessed and Shapiro–Wilks tests for normality carried out to assess if a spectrum of behavior traits was obtained. Shelter departure latency, area of enclosure explored and time spent active for those fish that left the shelter were checked for correlation using the Spearman coefficient to assess the relationship between each variable. A Welch two sample *t* test was carried out to compare the difference in lengths of fish that either did or did not leave the shelter to assess for size bias.

#### 2.7.1 Repeatability of behavior trials

The data obtained from the repeated trials were compared against data from the original trials using the “irr” package in R (Gamer et al., 2019). Cohen's Kappa test was used to compare repeatability of whether brown trout left the shelter or not between the two trials, providing a value of agreement between two scores from the same fish. Shelter departure latency, area of enclosure explored and time spent active were all compared between the two trials using Intraclass Correlation Coefficient (ICC), a standardized and well‐established ANOVA procedure for identifying repeatable genetic and behavioral traits (Lessells & Boag, 1987).

#### 2.7.2 Principal Component Analysis

Due to behavioral traits being correlated (Table 2), a Principal Component Analysis (PCA) was conducted on a correlation matrix to assign and collapse behavior variables (of those fish that had left the shelter) into behavioral trait dimensions, thereby reducing the number of variables used in the statistical analyses (Cote et al., 2011; Quinn & Keough, 2002). Principal Component Analysis outputs were assessed using scree plots identifying a change in slope (from steep to shallow) in the reported eigenvalues for each Principal Component (PC) thereby examining the variation explained by each PC group, and the eigenvalue greater than one rule was used where those PC groups with eigenvalues >1 were retained (Norman & Streiner, 1994; Quinn & Keough, 2002). Within PC groups that were retained, those behavior traits with a loading score >|0.4| were deemed to contribute to a PC group (behavioral trait dimension; Guadagnoli & Velicer, 1988).

**TABLE 2:** Spearman's rank correlations of behavior variables

Behavior variable	Area of enclosure explored	Time spent active
Shelter departure latency	−0.25, *p* = .09	−0.13, *p* = .38
Area of enclosure explored	—	0.51, *p* < **.001**

Note

Sample size of all variables is 49 fish.

#### 2.7.3 Passage behavior

All models described included only fish that behavior scores were available for, unless otherwise stated. Fish were deemed to have attempted passage of the bypass when first detected on A2 and succeeded when first detected on A5. Proportions of released fish that attempted passage, and proportions of attempting fish that succeeded in passage, were calculated for all fish. Generalized linear models with a binomial distribution were created to model passage success, which included only those fish that attempted passage. Passage success (*n* = 46; either “0” for fish that did not succeed or “1” for fish that did succeed in passing the weir) was modeled against all possible combinations of fish length and the values of each retained PC group outputted from the PCA analysis (Table 3). River discharge at time of last detection on A2 could not be used in the model due to fish with behavior scores failing in passage only at a time of elevated flow (only one elevated flow event occurred during the study period, otherwise flow conditions remained stable; passage attempts were seen throughout the study period), and thereby resulting in an almost perfect split of the data (such that during high flow conditions only failed passage attempts occurred). Further passage success models were, therefore, created which included all released fish that were also detected on A2 (*n* = 72) to identify whether river discharge may have predicted passage success (Table 3). Explanatory variables within these models were river discharge at time of last detection on A2, length of fish and the shelter departure latency score derived from behavioral trials (as each fish investigated was provided a score it seemed pertinent to include this variable to provide an indication of the role boldness has on passage success). As before, all possible combinations of these variables were trialed.

**TABLE 3:** Summary of analyses performed to analyze passage behavior

Question	Analysis (Sample size)	Data used
Passage success	Generalized linear model with Binomial distribution (*n* = 46)	Fish length + *Boldness btd* (PC2) + *Exploration‐Activity btd* (PC1)
Passage success	Generalized linear model with Binomial distribution (*n* = 72)	Fish length + Shelter Departure Latency + River Discharge
Time until First Attempt	General linear model (*n* = 46)	Fish length + *Boldness* *btd* (PC2) + *Exploration‐Activity btd* (PC1) + River Discharge
Passage Duration	General linear model (*n* = 46)	Fish length + *Boldness btd* (PC2) + *Exploration‐Activity btd* (PC1)
Number of attempts	Generalized linear model with Poisson distribution (*n* = 49)	Fish length + *Boldness* *btd* (PC2) + *Exploration‐Activity btd* (PC1)

The time taken from release until first detection on A2 (Time until First Attempt; *n* = 46) was modeled against all combinations of length of fish, river discharge at time of first detection on A2 and the values of each retained PC group outputted from the PCA analysis using general linear models (Table 3). Initial model testing identified that the residuals were not normally distributed, and so Time until First Attempt values were log transformed resulting in normalized residuals. Passage Duration for each fish was defined as the time interval between the last detection on A2 and the first detection on A5. General linear models were created to investigate Passage Duration (*n* = 46), with the values of each retained PC group outputted from the PCA analysis, length of fish and river discharge at time of last detection on A2 included (Table 3). All combinations of variables were trialed. After initial model testing, model residuals were identified as not being normally distributed, and so passage duration was inversely (reciprocal) transformed, resulting in normalized residuals. In both sets of Time until First Attempt and Passage Duration models, no correlated variables were included in the same model during model construction.

A new passage attempt was assumed to have been made after a lapse in time between two subsequent detections on A2 by an individual that was greater than 400 s. This was deemed an acceptable time for a passage attempt to have been made and a new attempt to have begun, based on calculating the gaps (time difference) between successive brown trout detections on PIT antennas within the bypass, plotting the log frequency of those gaps (binned in 20 s increments), and identifying the inflection point on the plot (here identified as the first gap of greater than 20 s where no detections were observed; Sibly et al., 1990; Castro‐Santos & Perry, 2012). The number of passage attempts made by all individuals (including those that did not attempt) that were included in the PCA analysis (i.e., those that left the shelter; *n* = 49) was modeled against the values of each retained PC group outputted from the PCA analysis and fish length using Poisson distributed generalized linear models. Again, all combination of variables were trialed (Table 3). Overdispersion of the models was checked for and not found.

Model selection for all models generated was based on minimizing Akaike's An Information Criterion (AIC). All models within ΔAIC = 6 were retained, and these were further refined by removing more complex models which had higher AIC values than simpler, nested counterparts (Richards, 2008). Therefore, a model containing three variables (e.g., A, B, and C) which had a lower AIC than a simpler model with only two of the same variables (e.g., A and B) was retained. However, if the more complex model had a greater AIC than a simpler nested model with the same variables, then the more complex model was rejected. Significant variables within each selected candidate model were identified as those where the 95% confidence interval of the coefficient estimates did not cross 0.

Any fish recaptured during electrofishing upstream of the weir (*n* = 24) were rereleased 60 m downstream of the weir to assess repeatability of passage behavior. These comprised the 12 fish that were recaptured on the 4 and 5 September for reassaying behavioral traits and 12 further fish that were recaptured during the initial experimental period. This provided a further investigation into behavior traits (in this case a form of activity behavior in homing) and also allowed for data to be gathered on the relationship between behavioral traits and the cumulative impact of multiple passages, which, due to the heavy fragmentation of rivers in Europe, occurs regularly in fish populations (Jones et al., 2019; Thorstad et al., 2008; Tummers et al., 2016). The Time until First Attempt and the Passage Duration between the first release and second release groups was compared using ICC to assess repeatability of passage behaviors. The difference in Time until First Attempt (Δ Time until First Attempt) and the difference in Passage Duration (Δ Passage Duration) between the two release groups was calculated. Separate Kendall‐Theil Sen Siegel (KTSS) non‐parametric linear regressions were generated to identify whether a relationship existed between Δ Time until First Attempt and the PC scores, and Δ Passage Duration and the values of each retained PC group outputted from the PCA analysis.

## 3 RESULTS

### 3.1 Behavior trials

In total, 78 brown trout were assayed in the experimental arena and PIT tagged. Length of brown trout ranged from 120 to 215 mm, with a mean ± *SD* = 146 ± 19 mm (Table 1). Of those 78, 49 brown trout left the shelter and were behaviorally typed. There was no difference in length between those that left the shelter (146 ± 16 mm) and those that did not (148 ± 24 mm; *t*_43.3_ = 0.52, *p* = .60).

The frequency of shelter departure latency behaviors was found to be bimodal, with 14 individuals leaving within 100 s, and 29 never leaving and being given the ceiling value of 900 s (mean ± *SD* = 561 ± 345 s; range = 1–900 s). A bimodal distribution of behaviors was also seen when those fish that did not leave the shelter were removed from the analysis, with peaks of leaving the shelter at 106 s and 614 s after the shelter door was removed (mean ± *SD* = 360 ± 284 s; range =1–900 s; Shapiro–Wilks: *w* = 0.90, *p* < .001). Of those that left the shelter, a range of the area of enclosure explored (mean ± *SD* = 2,508 ± 1,153 cm^2^; range = 704–4,864 cm²) and time spent active (mean ± *SD* = 211 ± 113 s; range = 11–506 s) behaviors were observed, with some individuals showing high levels of exploration and of activity, and others showing very low levels, being inactive for almost the entire experimental duration. The frequencies of area of enclosure explored (Shapiro–Wilks: *w* = 0.96, *p* = .11) and time spent active (Shapiro–Wilks: *w* = 0.98, *p* = .43) behaviors seen during the behavior trials were normally distributed, and both were found to be correlated. Shelter departure latency was not found to be significantly correlated with the other two behavior traits (Table 2).

Of the twelve brown trout that were successfully recaptured and recorded in the experimental arena a second time, seven exhibited the same behavior in leaving (*n* = 4) or remaining (*n* = 3) in the shelter, indicating 58.3% agreement to the original trials (Kappa = 0.17, *p* = .56). However, the shelter departure latency scores attributed to each of the 12 fish had a low repeatability estimate (ICC = 0.0, Table 4), suggesting that shelter departure latency is almost random within this sample group. Only four brown trout left the shelter in both trial sessions and enabled paired behavioral typing for area of enclosure explored and time spent active. Although area of enclosure explored was repeatable between the two groups (ICC = 0.4), time spent active was not (ICC = 0.0), again suggesting almost complete randomness in activity traits (Table 4).

**TABLE 4:** Results of intraclass correlation coefficient (ICC) for repeatability of each behavior trait from repeated behavior trials with brown trout

Variable	ICC Estimate	*F* (*df*)	*p*	No. brown trout
Shelter departure latency	0.0	0.7 (11,12)	.74	12
Area of enclosure explored	0.4	2.4 (3,4)	.21	4
Time spent active	0.0	0.4 (3,4)	.78	4

**FIGURE 3:**
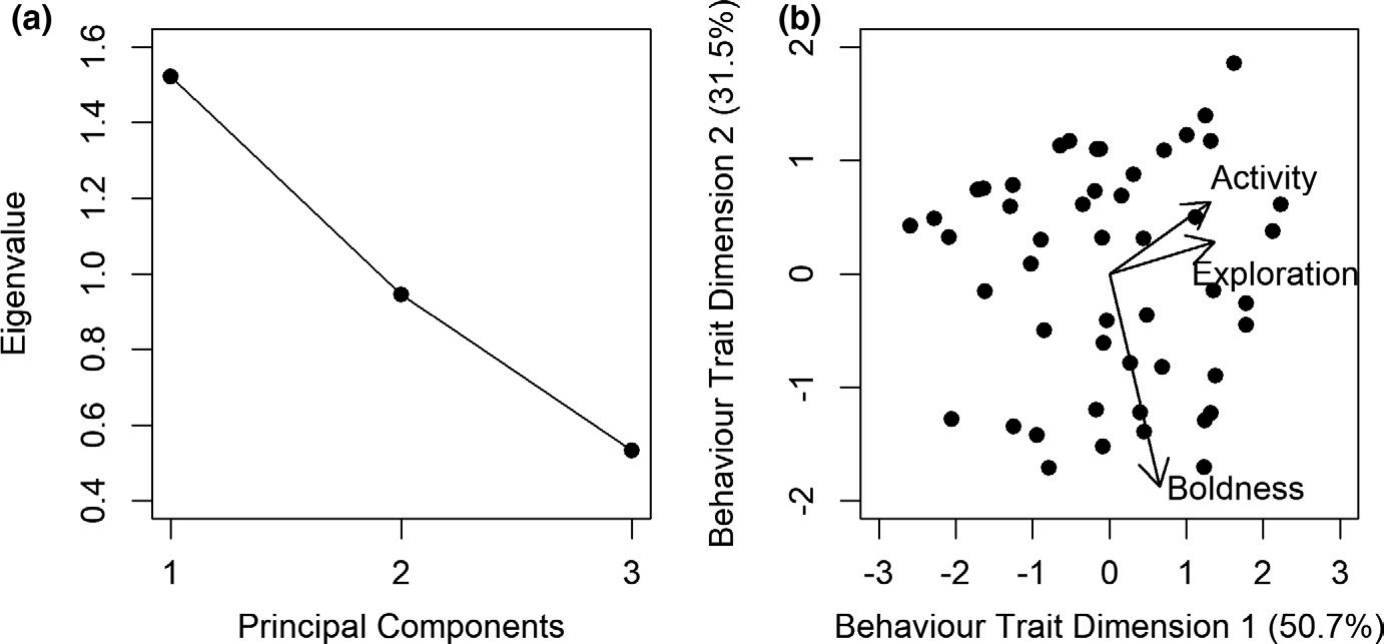
Scree plot of the principal components outputted by the principal component analysis (PCA; a) and biplot of the retained principal components (behavior trait dimensions) identifying the behavior trait groupings (b) for brown trout. Boldness, exploration, and activity refer to shelter departure latency, area of enclosure explored and time spent active, respectively

The PCA indicated three PC groups explaining 100.0% of the variation. Based on selection criteria, two PC groups explaining a total of 82.2% variation were retained (Table 5; Figure 3). Although the eigenvalue for the second PC group was lower than 1 (eigenvalue = 0.94; Table 5), the scree plot indicated it should be retained, with the proportional variance explained equaling 31.5%. The first PC group (50.7% of variation) consisted of area of enclosure explored and time spent active, thereby generating an exploration‐activity behavior trait dimension (hereafter referred to as *exploration‐activity btd*), with values ranging from −2.59 (“inactive” i.e., explored less of the enclosure and was spent less active) to 2.23 (“active” i.e., explored more of the enclosure and spent more time active; Figure 3). The second PC group consisted solely of shelter departure latency and thus generated a boldness behavior trait dimension (hereafter referred to as *boldness btd*), with values ranging from −1.71 (“shy” i.e., long shelter departure latency) to 1.86 (“bold” i.e., short shelter departure latency; Figure 3).

**TABLE 5:** Loading scores of behavior variables inputted into Principal Component Analysis, and the associated eigenvalues and proportional variance of each Principal Component (Behavior Trait Dimension [*btd*]) for behavior assays in brown trout

Variable	*Exploration‐Activity btd*	*Boldness btd*
Shelter departure latency	0.32	**−0.94**
Area of enclosure explored	**0.68**	0.14
Time spent active	**0.66**	0.32
Eigenvalue	1.52	0.94
Proportional variance (%)	50.7	31.5
Cumulative variance (%)	50.7	82.2

Note

Loading scores > |0.4| are given in bold.

### 3.2 Passage success and behavior

**FIGURE 4:**
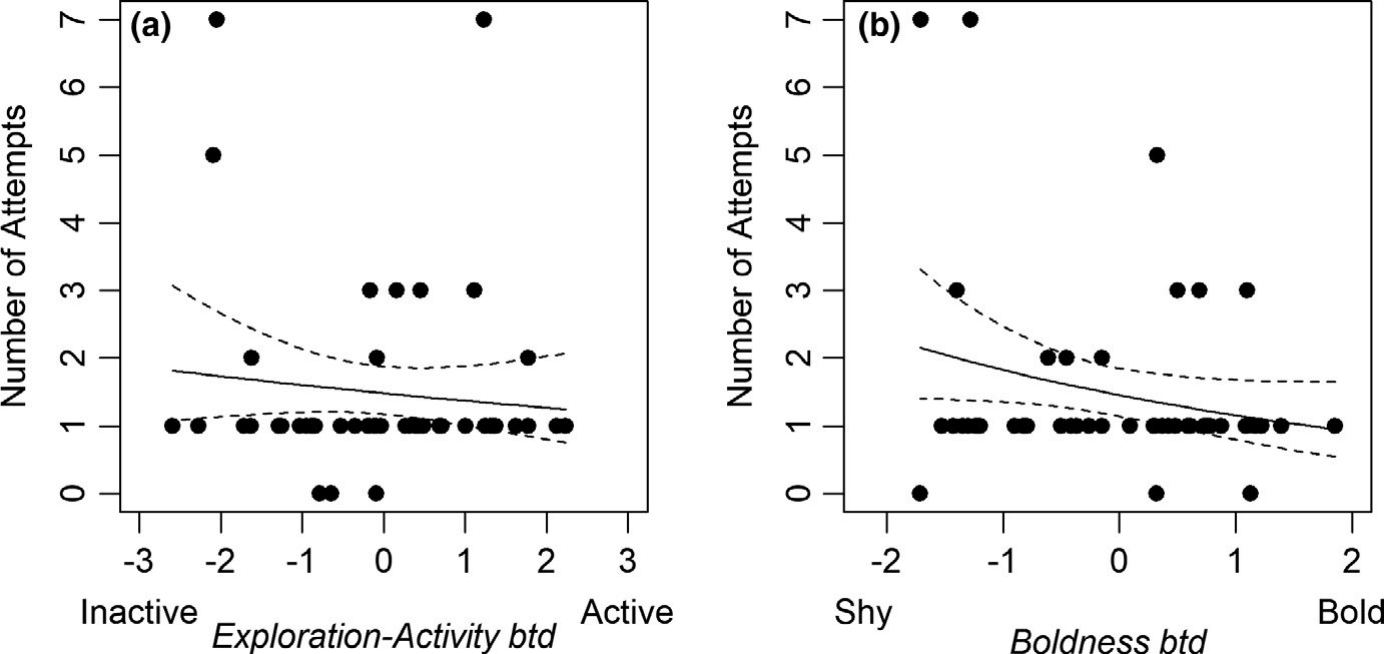
The relationship between the time spent active in the test enclosure (s; a) and the shelter departure latency (s; b) with the number of attempts made by individual brown trout. 95% confidence interval provided as dashed line

Throughout the study, only one of 78 brown trout left downstream through A1. In total, 72 (92.3% of the 78 released) attempted upstream passage via the bypass channel. This consisted of 46 brown trout that had left the shelter in the experimental arena (93.9% of 49 brown trout), and 26 that had not (89.7% of 29 brown trout). The median number of attempts exhibited by all brown trout (*n* = 78) and for those with behavior scores (*n* = 49) was one (range = 0–8) and one (range = 0–7), respectively. For those 49 fish with scored behavior traits, *Boldness btd* was retained within the best candidate model for explaining the number of attempts made by individual brown trout (Table 6). Although *Boldness btd* was not significant within the model, it showed a negative relationship with the number of attempts made by individual fish (Figure 4). Similarly, *Exploration‐Activity btd* also showed a negative relationship with number of attempts made, although *Exploration‐Activity btd* was not retained in any candidate model (Figure 4).

**TABLE 6:** Output of the generalized linear models created for explaining Time until First Attempt (*n* = 46), the number of attempts made by individual brown trout (*n* = 49), passage success (*n* = 46), and Passage Duration (*n* = 46) for those fish with behavior trait scores, with the coefficient estimates (and 95% confidence interval) for the independent variables retained in the each model provided

Model	AIC	ΔAIC	*df*	Intercept	Length	*Boldness btd*	*Exploration‐Activity btd*	River discharge
Number of attempts								
1	150.3	0	2	**0.37 (0.13, 0.60)**		−0.23 (−0.46, 0.01)		n/a
2	152.0	1.7	1	**0.40 (0.16, 0.61)**				n/a
Time until First Attempt								
1	139.8	0	4	**−4.33 (−7.17, −1.49)**	0.02 (−0.0004, 0.0376)			**5.72 (3.73, 7.70)**
2	141.8	2	3	**−1.62 (−2.22, −1.03)**				**5.70 (3.65, 7.74)**
Passage success								
1	18.5	0	1	**3.09 (1.92, 4.90)**				n/a
Passage Duration								
1	−227.7	0	3	−0.01 (−0.06, 0.03)	**0.0004 (0.0001, 0.0004)**			
2	−222.6	5.1	2	**0.05 (0.04, 0.05)**				

Note

All candidate models within 6 ΔAIC are reported. Significant variables are given in bold. n/a denotes variable not included in the analyses for that model.

**FIGURE 5:**
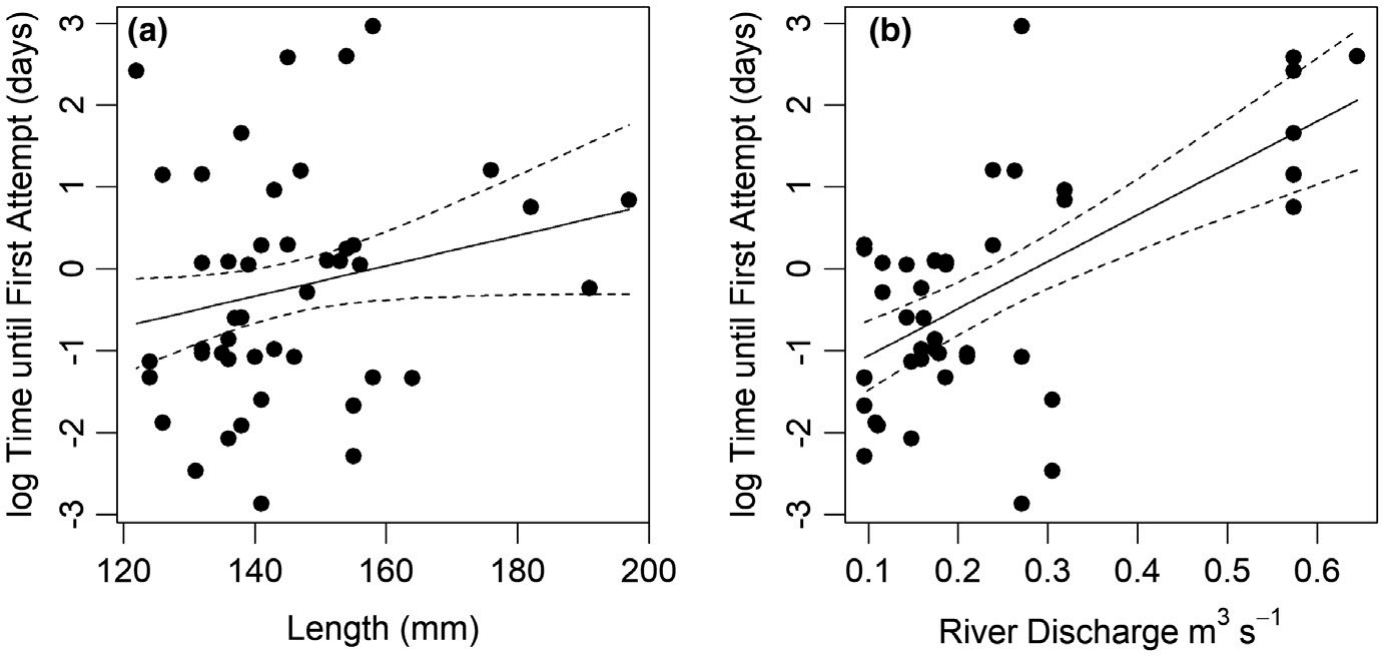
The relationship between brown trout length (mm; a) and river discharge (m^3^/s; b) with the time taken from release until first attempt (days) log‐transformed. 95% confidence interval provided as dashed line

The median (25th, 75th percentile) Time until First Attempt was 1.05 (0.31, 2.32) days. The best candidate model explaining Time until First Attempt for the 46 fish that attempted retained fish length and river discharge (Table 6). River discharge had a significant positive relationships in the model (Figure 5), and although fish length was not significant, it also showed a positive relationship with Time until First Attempt (Figure 5). *Boldness btd* and *Exploration‐Activity btd* were not retained in any candidate model.

**FIGURE 6:**
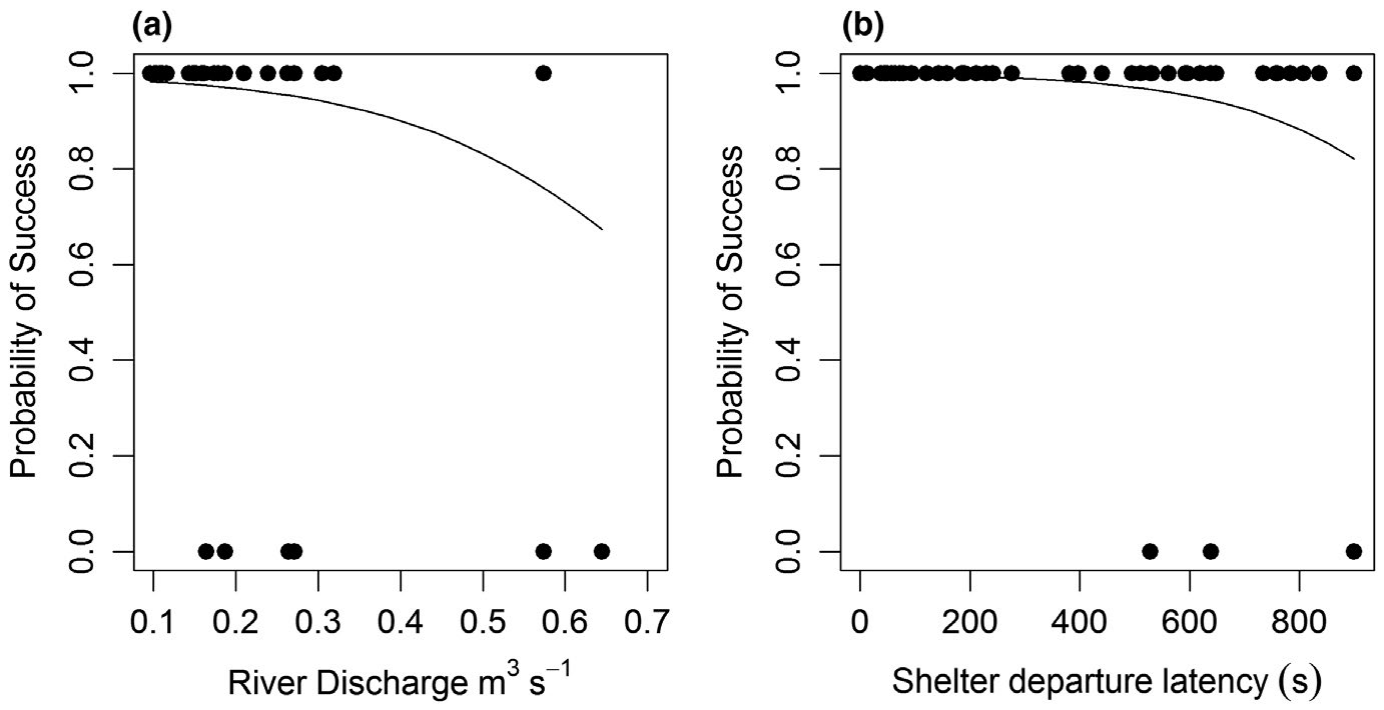
The relationship between river discharge (a) and shelter departure latency (s; b) and the probability of passage success for brown trout

Sixty‐five (90.3% of the 72 that approached) brown trout succeeded in passing upstream using the bypass channel, consisting of 44/46 (95.7%) that had left the shelter, and 21/26 (80.8%) that had not. No variables were found to be significant in any candidate models explaining passage success for the 46 brown trout that had left the shelter (Table 6). The best candidate model was the empty model with no variables. When modeling passage success for all 72 fish that had attempted passage regardless of whether they had left the shelter or not, the ΔAIC identified the best candidate model retained river discharge and shelter departure latency (Table 7). Shelter departure latency was found to be significantly negatively related (estimate = −0.004; Figure 6) and was considered an important variable as it was the only variable retained in the next best candidate model within 3.4 ΔAIC of the best candidate model. River discharge was also found to be significantly negatively related to passage success probability (estimate = −6.03; Figure 6a).

**TABLE 7:** Output of the generalized linear model created for passage for all fish (*n* = 72), with the coefficient estimates (and 95% confidence interval) for the independent variables retained in the each model provided

Model	AIC	ΔAIC	*df*	Intercept	Length	Shelter departure latency	River discharge
1	41.2	0	3	**7.36 (3.66, 14.28)**		**−0.005 (−0.012, −0.001)**	**−6.03 (−11.80, −1.01)**
2	44.6	3.4	2	**4.79 (2.33, 9.69)**		**−0.004 (−0.009, −0.0004)**	
3	46.5	5.3	2	**3.37 (1.94, 5.17)**			−4.2 (−8.57, 0.27)

Note

All candidate models within 6 ΔAIC are reported. Significant variables are given in bold.

The best candidate model describing Passage Duration for the 46 brown trout with behavior measures that attempted passage included brown trout length only, with larger brown trout taking longer to traverse the bypass. The next best candidate model was the empty model with 5.1 ΔAIC of the best candidate model (Table 6). Neither *Boldness btd* nor *Exploration‐Activity btd* were retained in any candidate model, nor was river discharge (Table 6).

### 3.3 Behavior changes from multiple passages

Twenty‐four fish (including the 12 reassayed fish) were recaptured throughout the study period and rereleased downstream of the weir. Twenty‐one (87.5%) of these fish were detected attempting and succeeding in passage for a second time. Repeatability estimates of 0.2 (*F*_20,21_ = 1.4, *p* = .22) and 0.5 (*F*_20,21_ = 2.6, *p* = .02) were observed between the two releases of the 21 fish for Time until First Attempt and Passage Duration, respectively. In general, fish were first detected on A2 quicker after the second release (median [25th, 75th percentile], 0.41 [0.25, 1.14] days) than after the first release (0.59 [0.26, 2.13] days), and also exhibited shorter Passage Duration after the second release (median [25th, 75th percentile], 17.75 [11.53, 23.60] min) than after the first release (21.10 [15.57, 27.36] min).

**FIGURE 7:**
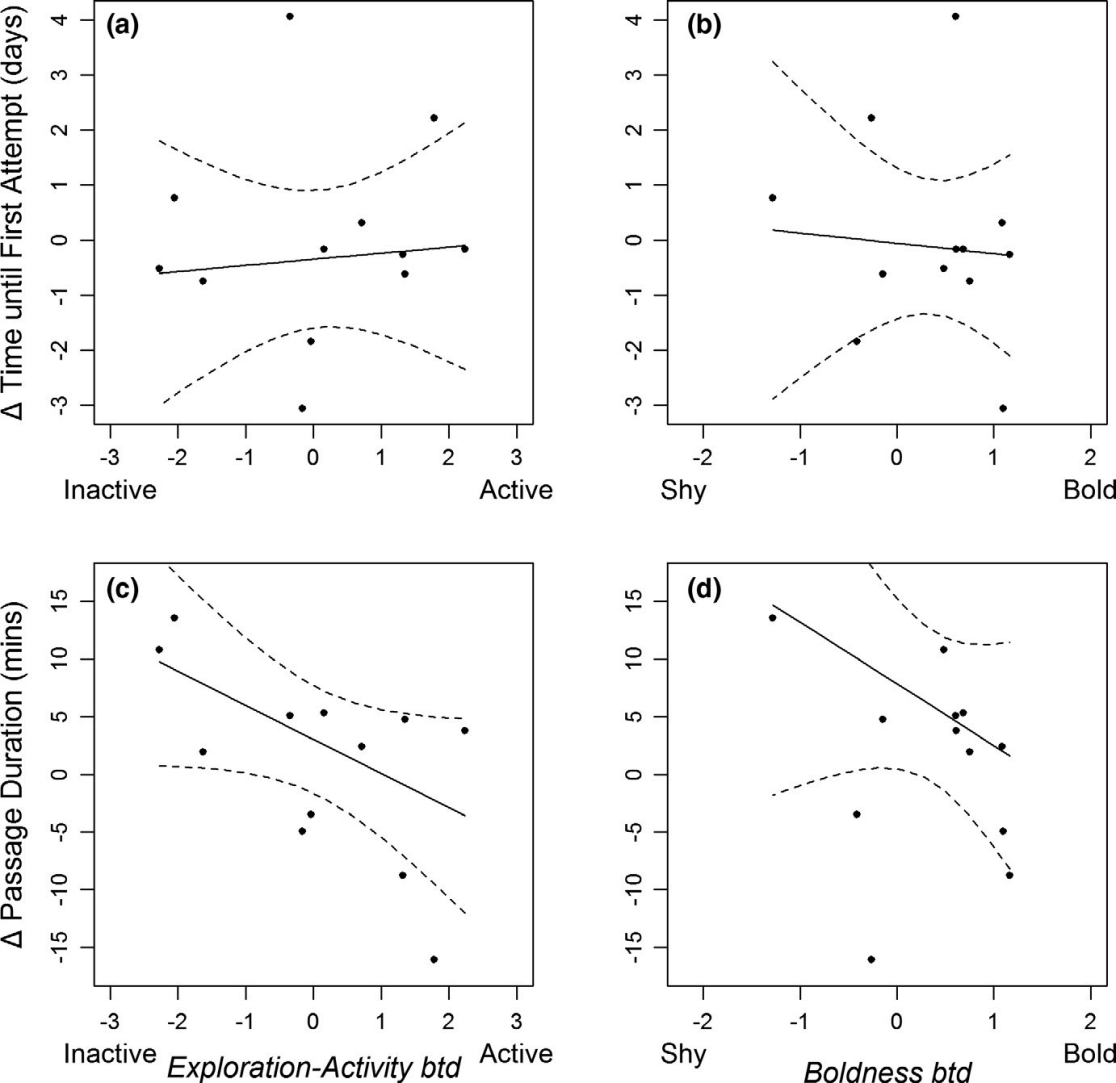
Relationship between the Δ Time until First Attempt in the first and second release groups of brown trout, and *Exploration‐Activity btd* (a) and *Boldness btd* (b), and the relationship between Δ Passage Duration in the first and second release groups, and *Exploration‐Activity btd* (c) and *Boldness btd* (d). 95% confidence interval provided as dashed line

Thirteen of the 24 re‐released fish had behavior scores from the first behavior assay, and 12 (92.3%) of these fish attempted and succeeded in passing the weir. All fish passed the weir after only one attempt (number of attempts of these fish during first release ranged from 1 to 8). No relationship was found between Δ Time until First Attempt and either *Exploration‐Activity btd* (KTSS, *V*_1,10_ = 57, *p* = .17; Figure 7a) or *Boldness btd* (KTSS, *V*_1,10_ = 25, *p* = .29; Figure 7b).

For the 12 fish with behavior scores and that also passed the weir after both releases, Δ Passage Duration was found to be significantly, negatively related to both *Exploration‐Activity btd* (KTSS, *V*_1,10_ = 2, *p* = .004; Figure 7c) and *Boldness btd* (KTSS, *V*_1,10_ = 12, *p* = .03; Figure 7d). This suggests that those individuals that explore less, are less active and shyer, pass the weir more slowly during the second passage relative to the first passage.

## 4 DISCUSSION

### 4.1 Implications of behavior traits on passage

Passage success in this study was higher than previously reported for translocated brown trout at this weir and nature‐like bypass (this study: 90.3% of all 72 fish that attempted, 95.7% of the 46 fish included in the PCA and that attempted; Tummers et al., 2016: 70.2% of fish that attempted). Across all 72 fish that attempted passage, shelter departure latency was retained in the best candidate passage success model for all fish, suggesting that bolder individuals (i.e., those that left the shelter quicker) had a greater probability of succeeding in passage than shyer fish. This supports the predictions of Hirsch et al. (2017) and findings of Jones et al. (2021), but contrasts with the findings reported by Landsman et al. (2017) who showed no relationship between boldness and passage success. However, when reducing the sample size to only those that had left the shelter and thus included in the PCA to obtain behavioral trait dimensions, then *Boldness btd* was not retained by any candidate passage success model. Similarly, *Exploration‐Activity btd* was not retained by any candidate passage success model. This may have occurred for two reasons. Firstly, passage success may not be dependent on fish behavioral traits, but as a greater sample size showed a relationship for boldness, this is unlikely, though such a relationship would still be weak. Secondly, the sample size in this study was too small to identify relationships, particularly when excluding those fish that had not left the shelter.

No behavioral trait was retained in any candidate models describing Time until First Attempt or Passage Duration, indicating that the behavioral trait dimensions identified do not influence either of these passage metrics. *Exploration‐Activity btd* was also not retained in any candidate model describing the number of passage attempts made, despite the prediction that those fish that explore more and are more active would require fewer passage attempts. Again, this may be an artifact of the sample size which might limit differences in any of these passage metrics being identified. Furthermore, as the majority of brown trout only took one attempt to pass through the fishway and there were few fish that never attempted, perhaps the nature‐like bypass was not a difficult enough obstacle to demonstrate differences in behavioral traits for the sample size used. Nature‐like bypasses are designed to mimic a river's natural hydraulic complexity, with a maximum slope of 2% and natural features throughout (Jungwirth et al., 1998), making it a realistic extension of the river. Therefore, these results could suggest that nature‐like bypasses similar to that studied do not favor certain behavioral types, and as such, should be the preferred fishway type for brown trout, in order to increase longitudinal connectivity in rivers, since they are also passable by a wide range of fish sizes and in a wide range of conditions (Dodd et al., 2017; Tummers et al., 2016). However, activity and exploration behavioral traits have not been found to drive dispersal in mosquitofish (*Gambusia affinis*; Cote et al., 2011), nor were they found to explain movement patterns of common voles (*Microtus arvalis*) in habitat corridors (Kowalski et al., 2019). Therefore, these two behavioral traits (exploration and activity) may not contribute substantively to a native species in gaining access to further habitat.

*Boldness btd* was the only variable retained in the best candidate model describing the number of passage attempts made, suggesting that bolder individuals required fewer attempts. Although this was not significant, the 95% confidence interval was very close to not passing 0, suggesting a larger sample size might have refined the confidence interval range. If bolder individuals do require fewer passage attempts to pass upstream, this might have several consequences. Firstly, more passage attempts at passage bottlenecks, such as at barriers, would likely increase exposure to predators (Agostinho et al., 2012; Alcott et al., 2020; Schwinn et al., 2018). Secondly, bolder fish might have greater upstream access to underutilized resources, such as food‐rich habitat patches, than shy fish, potentially resulting in differential growth, though complicated by habitat complexity and fish activity effects (Adriaenssens & Johnsson, 2011; Höjesjö et al., 2004). In our study system, it is unlikely that the relatively small increase in passage attempts by shy fish had marked effects on their energy expenditure, but if similar shy‐bold passage effects occur for upstream‐migrating adults at larger obstacles distant from spawning habitat, such impacts could be substantial and deserve study. Although ontogenetic shifts in behavioral traits have been postulated through individuals gaining experiences, and particularly in those with complex life‐history strategies (Sih & Bell, 2008), in diadromous fishes, for example, their exposure to fishways during development are limited to downstream migration, and so therefore their encounters with fishways, regardless of nature‐like or technical design, during upstream migration may be novel, and thus fish may not have gained the experience to alter their behavior traits in response to this interaction.

As a result of the extensive modifications made to rivers by humans, high densities of barriers to movements means fish potentially have multiple obstacles to traverse during riverine movements, particularly for migratory fishes (Jones et al., 2019; Thorstad et al., 2008; Tummers et al., 2016). In Great Britain, an estimated 97% of the river network is subject to fragmentation, and there is a national artificial barrier density of 0.27 barriers per river km (Jones et al., 2019). The cumulative impact of weirs on fish movement has not been fully investigated. However, a study on the River Deerness (which contained seven barriers to movement), Northeast England, showed that only three of 30 radio‐tracked adult brown trout that had entered the Deerness were detected upstream of the sixth barrier and none upstream of the seventh (Tummers et al., 2016). Another study carried out across a 15‐year period in the River Nivelle, Northern Basque Country (France/Spain), identified that Atlantic salmon nest aggregations were greatest immediately downstream of weirs, suggesting that weirs constrained the distribution of Atlantic salmon through the river (Tentelier & Piou, 2011). Although passage success in the current study for trout that were recaptured and released a second time was 100% (*n*_passed/attempting/released_ = 21/21/24), arguably all fish should have passed as they had already successfully passed the weir prior to the second release. Passage behaviors post‐second‐release were analogous to those seen in the post‐first‐release, with all fish passing on the first attempt, and faster and largely repeatable (within the values reported by Bell et al. (2009) for active behaviors) Time until First Attempt and Passage Duration.

Importantly, the Δ Passage Duration was found to be significantly influenced by both *Boldness btd* and *Exploration‐Activity btd*, with shyer and more inactive fish passing the weir slower on the second passage than the first, and bolder and more active fish passing quicker on the second passage. This relationship was not seen in Δ Time until First Attempt, but may again be a result of small sample size (*n* = 12). The influence of these behavioral traits on the passage behaviors exhibited at consecutive passage attempts may have major ramifications, where shyer and more inactive individuals may increase their exposure to predation by being slower in passing (Thorstad et al., 2008).

### 4.2 Behavioral typing

Boldness, exploration, and activity behavior traits were obtained from open‐field experiments and collapsed into two behavioral trait dimensions: *Exploration‐Activity btd* and *Boldness btd*. Data obtained from the boldness behavior trials (shelter departure latency) showed similar bimodal frequencies as reported in the literature (Landsman et al., 2017), and the observed area of the enclosure explored and time spent active were normally distributed, indicating that the behaviors seen reflect those which could be expected. The two behavior trait dimensions produced from the PCA are also consistent with those previously reported (Cote et al., 2010, 2011). It may be debated whether behavioral trait measurement should be carried out before or after tagging; in this study, we chose to tag after behavioral trait measurement because tagging required anesthesia under animal welfare certification, the effects of which on behavioral trait measurement are unclear. We highlight the need for a well‐controlled laboratory study examining the effects of anesthesia, handling, and tagging on behavioral trait measurement in fishes.

Unfortunately, there was a lack of repeatability in the behavioral traits measured in this study (range 0–0.4), indicating personality was not measured, but instead identifying behavioral trait dimensions. Firstly, the low sample size used in the repeatability analyses for this study cannot be dismissed, and lower sample sizes are known to result in lower repeatability estimates (Bell et al., 2009; Dingemanse & Dochtermann, 2013). Another argument could be made that the time between the first and second observation for retrialed fish varied too much (range = 13–41 days) and that repeatability estimates decrease with increased time between measurements (Bell et al., 2009). This decrease in repeatability is driven by maturity and different requirements between different life stages or sampling periods (Bell et al., 2009). Although the fish used in this study were all juvenile (estimated to be between 1 and 2 years old), sexual maturation of “juvenile” brown trout has been observed in this system from late‐September (but with a peak in late‐October/early‐November; A. Lothian, personal observation). The second behavioral typing occurred in early‐September, shortly before juveniles have been observed to be sexual mature in this system, and despite no individuals being seen to be sexually mature at time of retrials, it cannot be ruled out that some of these individuals may have been undergoing sexual maturation at time of second sampling, thus exhibiting an altered behavioral trait.

Throughout the study period (August 2018), tagged fish were caught within the ~550 m stretch of river upstream of the weir, beginning at the weir and finishing where initial sampling had ended. Despite this, in early‐September 2018, only 12 tagged fish were caught. Although predation pressures are high in the Deerness for juvenile brown trout through the presence of grey heron (*Ardea cinerea*), otter (*Lutra lutra*), and American mink (*Neovison vison*), there was no evidence for a reduction in the population size during sampling for fish to retrial. A similar abundance of brown trout was observed during both electrofishing periods (an experienced electrofishing team with >70% electrofishing capture efficiency for >1‐year‐old brown trout over first pass in this river). The placement of antenna A1 downstream of the weir identified that fish had not moved downstream either, and so it is likely that a substantial proportion of the missing trout moved upstream out of the study reach.

Another factor that might limit the repeatability of the study was that the behavior trials were carried out *in situ* in the river, where it is not possible to control the environment and isolate single behavioral traits. Under regular laboratory conditions, all elements of the study are standardized (Cote et al., 2011). However, the likes of external olfactory and visual cues could not be removed in this study. In video footage, small shoals of little fish (likely young minnow [*Phoxinus phoxinus*]) were seen entering the enclosure during trials, with no ability to identify how this influences behavioral traits exhibited by brown trout (no reactions were seen by the brown trout, but that does not mean that the presence of these smaller fish did not influence behavior). Furthermore, although there are no fish in this river that act as predators which might influence behaviors were they to pass nearby the enclosure, American mink were seen in the area between the trial site and the weir during behavior trials. The presence of this predator could have encouraged other free‐ranging brown trout in the river to release predatory alarm cues, which have been seen to reduce activity and exploratory behaviors in conspecifics when tested under laboratory conditions (Kopack et al. 2015; Mirza & Chivers, 2003).

However, a review of 759 papers by Bell et al. (2009) on repeatability estimates of behavioral traits identified that activity behavior traits are among the most unrepeatable across all taxa (mean effect size in the literature ~0.25; effect size in this study −0.5). Exploratory behaviors, on the other hand, were reported to have higher repeatability in the literature, with effect sizes of ~0.5, similar to that seen in this study (effect size of 0.4). Therefore, the repeatability scores in this study were found to be within the range reported within the literature. Another key finding of the Bell et al. (2009) review that is pertinent to this study was that ectotherms exhibit significantly lower repeatability across all behavior traits than endotherms, presumably due to differences in the reliance on environmental conditions that drive behaviors. In addition, fishes exhibited the second lowest mean amalgamated repeatability estimates (~0.25) across all taxa reported (Bell et al. 2009). Although personality was not identified within this experiment due to the lack of repeatability and consistent individual differences, behavioral traits that could be collapsed onto behavioral trait dimensions were reliably identified.

### 4.3 Conclusion

Intraspecific variation in behavioral traits (but not consistent individual differences, or personality) was identified in juvenile brown trout using a novel approach of measuring behavior *in situ* within the river. Boldness was retained in the model describing the number of attempts made by an individual and also found to significantly influence passage success, suggesting bolder individuals require fewer attempts and have an increased probability of succeeding in passage. Furthermore, boldness, exploration, and activity all had a significant influence on the change in passage behaviors observed at consecutive, successful passage attempts. This study, therefore, suggests that bold and active individuals may perform better during fishway passage attempts, particularly within rivers where multiple barriers to movement exist. However, it is also evident that more research is needed to confirm this and to investigate the relationship of any such variable with migration obstacle effects for downstream, as well as upstream migration.

## CONFLICT OF INTEREST

The authors declare no competing interests.

## AUTHOR CONTRIBUTIONS

**Angus J. Lothian:** Conceptualization; Formal analysis ; Investigation; Methodology; Project administration; Writing‐original draft; Writing‐review & editing. **Martyn C. Lucas:** Conceptualization; Methodology; Project administration; Resources; Supervision; Writing‐review & editing.

## Data Availability

Data have been available on DRYAD (https://doi.org/10.5061/dryad.bnzs7h4bn).

## References

[ece37964-bib-0001] Adriaenssens, B., & Johnsson, J. I. (2011). Shy trout grow faster: Exploring links between personality and fitness‐related traits in the wild. Behavioral Ecology, 22, 135–143. 10.1093/beheco/arq185

[ece37964-bib-0002] Agostinho, A. A., Agostinho, C. S., Pelicice, F. M., & Marques, E. E. (2012). Fish ladders: Safe fish passage or hotspot for predation? Neotropical Ichthyology, 10(4), 687–696. 10.1590/S1679-62252012000400001

[ece37964-bib-0003] Alcott, D., Long, M., & Castro‐Santos, T. (2020). Wait and snap: Eastern snapping turtles (*Chelydra serpentina*) prey on migratory fish at road‐stream crossing culverts. Biology Letters, 16, 20200218. 10.1098/rsbl.2020.0218 32961086 PMC7532709

[ece37964-bib-0004] Álvarez, D., & Bell, A. M. (2007). Sticklebacks from streams are more bold than sticklebacks from ponds. Behavioural Processes, 76, 215–217. 10.1016/j.beproc.2007.05.004 17583445

[ece37964-bib-0005] Andren, H. (1994). Effects of habitat fragmentation on birds and mammals in landscapes with different proportions of suitable habitat: A review. Oikos, 71(3), 355–366.

[ece37964-bib-0006] Armstrong, J. D., & Herbert, N. A. (1997). Homing movements of displaced stream dwelling brown trout. Journal of Fish Biology, 44, 445–449. 10.1111/j.1095-8649.1997.tb01372.x

[ece37964-bib-0007] Beben, D. (2016). Crossings construction as a method of animal conservation. Transportation Research Procedia, 14, 474–483. 10.1016/j.trpro.2016.05.100

[ece37964-bib-0008] Bell, A. M. (2007). Future directions in behavioural syndromes research. Proceedings of the Royal Society B: Biological Sciences, 274, 755–761. 10.1098/rspb.2006.0199 PMC1919401 17251088

[ece37964-bib-0009] Bell, A. M., Hankison, S. J., & Laskowski, K. L. (2009). The repeatability of behaviour: A meta‐analysis. Animal Behaviour, 77, 771–783. 10.1016/j.anbehav.2008.12.022 24707058 PMC3972767

[ece37964-bib-0010] Bell, A. M., & Sih, A. (2007). Exposure to predation generates personality in threespined sticklebacks (*Gasterosteus aculeatus*). Ecology Letters, 10(9), 828–834. 10.1111/j.1461-0248.2007.01081.x 17663716

[ece37964-bib-0011] Birnie‐Gauvin, K., Thorstad, E., & Aarestrup, K. (2019). Overlooked aspects of the *Salmo salar* and *Salmo trutta* lifecylces. Reviews in Fish Biology and Fisheries, 29, 749–766.

[ece37964-bib-0012] Brackley, R., Lucas, M. C., Thomas, R., Adams, C. E., & Bean, C. W. (2018). Comparison of damage to live v. euthanized Atlantic salmon *Salmo salar* smolts from passage through an Archimedean screw turbine. Journal of Fish Biology, 92(5), 1635–1644. 10.1111/jfb.13596 29537067

[ece37964-bib-0013] Brown, C., Jones, F., & Braithwaite, V. (2005). In situ examination of boldness‐shyness traits in the tropical poeciliid, *Brachyraphis episcopi* . Animal Behaviour, 70(5), 1003–1009. 10.1016/j.anbehav.2004.12.022

[ece37964-bib-0014] Brown, R. S., Eppard, M. B., Murchie, K. J., Nielsen, J. L., & Cooke, S. J. (2011). An introduction to the practical and ethical perspectives on the need to advance and standardize the intracoelomic surgical implantation of electronic tags in fish. Reviews in Fish Biology and Fisheries, 21(1), 1–9. 10.1007/s11160-010-9183-5

[ece37964-bib-0015] Bunt, C. M., Castro‐Santos, T., & Haro, A. (2012). Performance of fish passage structures at upstream barriers to migration. River Research and Applications, 28, 457–478. 10.1002/rra.1565

[ece37964-bib-0016] Castro‐Santos, T., Cotel, A., & Webb, P. (2009). Fishway evaluations for better bioengineering: An integrative approach. In A. J. Haro, K. L. Smith, R. A. Rulifson, C. M. Moffit, R. J. Klauda, M. J. Dadswell, R. A. Cunjak, J. E. Cooper, K. L. Beal, & T. S. Avery (Eds.), Challenges for diadromous fishes in a dynamic global environment (pp. 557–575). American Fisheries Society.

[ece37964-bib-0017] Castro‐Santos, T., & Haro, A. (2010). Fish guidance and passage at barriers. In P. Domenci, & B. G. Kapoor (Eds.), Fish Locomotion: An eco‐ethological perspective (pp. 62–89). Science Publishers.

[ece37964-bib-0018] Castro‐Santos, T., & Perry, R. (2012). Time‐to‐event analysis as a framework for quantifying fish passage performance. In N. S. Adams, J. W. Beeman, & J. H. Eiler (Eds.), Telemetry techniques: A user guide for fisheries research (pp. 427–452). American Fisheries Society.

[ece37964-bib-0019] Chapman, B. B., Hulthén, K., Blomqvist, D. R., Hansson, L. A., Nilsson, J. Å., Brodersen, J., Anders Nilsson, P., Skov, C., & Brönmark, C. (2011). To boldly go: Individual differences in boldness influence migratory tendency. Ecology Letters, 14, 871–876. 10.1111/j.1461-0248.2011.01648.x 21718420

[ece37964-bib-0020] Chapple, D. G., Simmonds, S. M., & Wong, B. B. M. (2012). Can behavioral and personality traits influence the success of unintentional species introductions? Trends in Ecology and Evolution, 27, 57–62. 10.1016/j.tree.2011.09.010 22001529

[ece37964-bib-0021] Conrad, J. L., Weinersmith, K. L., Brodin, T., Saltz, J. B., & Sih, A. (2011). Behavioural syndromes in fishes: A review with implications for ecology and fisheries management. Journal of Fish Biology, 78(2), 395–435. 10.1111/j.1095-8649.2010.02874.x 21284626

[ece37964-bib-0022] Cooke, S. J., Hinch, S. G., Lucas, M. C., & Lutcavage, M. (2012). Biotelemetry and biologging. In A. V. Zale, D. L. Parrish, & T. M. Sutton (Eds.), Fisheries techniques (3rd ed., pp. 819–881). American Fisheries Society.

[ece37964-bib-0023] Cote, J., Fogarty, S., Brodin, T., Weinersmith, K., & Sih, A. (2011). Personality‐dependent dispersal in the invasive mosquitofish: Group composition matters. Proceedings of the Royal Society B: Biological Sciences, 278(1712), 1670–1678. 10.1098/rspb.2010.1892 PMC3081767 21068033

[ece37964-bib-0024] Cote, J., Fogarty, S., Weinersmith, K., Brodin, T., & Sih, A. (2010). Personality traits and dispersal tendency in the invasive mosquitofish (*Gambusia affinis*). Proceedings of the Royal Society B: Biological Sciences, 277(1687), 1571–1579. 10.1098/rspb.2009.2128 PMC2871838 20071380

[ece37964-bib-0025] Dingemanse, N. J., & Dochtermann, N. A. (2013). Quantifying individual variation in behaviour: Mixed‐effect modelling approaches. Journal of Animal Ecology, 82, 39–54. 10.1111/1365-2656.12013 23171297

[ece37964-bib-0026] Dingemanse, N. J., Kazem, A. J. N., Réale, D., & Wright, J. (2010). Behavioural reaction norms: Animal personality meets individual plasticity. Trends in Ecology and Evolution, 25(2), 81–89. 10.1016/j.tree.2009.07.013 19748700

[ece37964-bib-0027] Dingemanse, N. J., & Réale, D. (2005). Natural selection and animal personality. Behaviour, 142, 1159–1184. 10.1163/156853905774539445

[ece37964-bib-0028] Dodd, J. R., Bolland, J. D., Hateley, J., Cowx, I. G., Walton, S. E., Cattaneo, M. E. G. V., & Noble, R. A. A. (2018). Upstream passage of adult sea trout (*Salmo trutta*) at a low‐head weir with an Archimedean screw hydropower turbine and co‐located fish pass. Marine and Freshwater Research, 69, 1822–1833. 10.1071/MF18125

[ece37964-bib-0029] Dodd, J. R., Cowx, I. G., & Bolland, J. D. (2017). Efficiency of a nature‐like bypass channel for restoring longitudinal connectivity for a river‐resident population of brown trout. Journal of Environmental Management, 204, 318–326. 28898753 10.1016/j.jenvman.2017.09.004

[ece37964-bib-0030] Duckworth, R. A. (2006). Behavioral correlations across breeding contexts provide a mechanism for a cost of aggression. Behavioral Ecology, 17, 1011–1019. 10.1093/beheco/arl035

[ece37964-bib-0031] Ferguson, A., Reed, T. E., Cross, T. F., McGinnity, P., & Prodöhl, P. A. (2019). Anadromy, potamodromy and residency in brown trout *Salmo trutta*: The role of genes and the environment. Journal of Fish Biology, 95(3), 692–718. 10.1111/jfb.14005 31197849 PMC6771713

[ece37964-bib-0032] Forty, M., Spees, J., & Lucas, M. C. (2016). Not just for adults! Evaluating the performance of multiple fish passage designs at low‐head barriers for the upstream movement of juvenile and adult trout *Salmo trutta* . Ecological Engineering, 94, 214–224. 10.1016/j.ecoleng.2016.05.048

[ece37964-bib-0033] Fraser, D. F., Gilliam, J. F., Daley, M. J., Le, A. N., & Skalski, G. T. (2001). Explaining leptokurtic movement distributions: Intrapopulation variation in boldness and exploration. The American Naturalist, 158(2), 124–135. 10.1086/321307 18707341

[ece37964-bib-0034] Gamer, M., Lemon, J., Fellows, I., & Singh, P. (2019). irr: Various coefficients of interrater reliability and agreement. https://cran.r‐project.org/web/packages/irr/ Version: 0.84.1

[ece37964-bib-0035] Greenberg, L. A. (1992). The effect of discharge and predation on habitat use by wild and hatchery brown trout (*Salmo trutta*). Regulated Rivers: Research & Management, 7(2), 205–212. 10.1002/rrr.3450070208

[ece37964-bib-0036] Guadagnoli, E., & Velicer, W. F. (1988). Relation of sample size to the stability of component patterns. Psychological Bulletin, 103(2), 265–275. 3363047 10.1037/0033-2909.103.2.265

[ece37964-bib-0037] Haddad, N. M., Brudvig, L. A., Clobert, J., Davies, K. F., Gonzalez, A., Holt, R. D., Lovejoy, T. E., Sexton, J. O., Austin, M. P., Collins, C. D., Cook, W. M., Damschen, E. I., Ewers, R. M., Foster, B. L., Jenkins, C. N., King, A. J., Laurance, W. F., Levey, D. J., Margules, C. R., … Townshend, J. R. (2015). Habitat fragmentation and its lasting impact on Earth's ecosystems. Science Advances, 1(2), 1–10. 10.1126/sciadv.1500052 PMC4643828 26601154

[ece37964-bib-0038] Haigh, A., O'Riordan, R. M., & Butler, F. (2014). Hedgehog *Erinaceus europaeus* mortality on Irish roads. Wildlife Biology, 20(3), 155–160. 10.2981/wlb.12126

[ece37964-bib-0039] Halvorsen, M., & Stabell, O. B. (1990). Homing behaviour of displaced stream‐dwelling brown trout. Animal Behaviour, 39(6), 1089–1097. 10.1016/S0003-3472(05)80781-X

[ece37964-bib-0040] Hertel, A. G., Niemelä, P. T., Dingemanse, N. J., & Mueller, T. (2020). A guide for studying among‐individual behavioral variation from movement data in the wild. Movement Ecology, 8(1), 1–18. 10.1186/s40462-020-00216-8 32612837 PMC7325061

[ece37964-bib-0041] Hirsch, P. E., Thorlacius, M., Brodin, T., & Burkhardt‐Holm, P. (2017). An approach to incorporate individual personality in modeling fish dispersal across in‐stream barriers. Ecology and Evolution, 7, 720–732. 10.1002/ece3.2629 28116066 PMC5243775

[ece37964-bib-0042] Höjesjö, J., Johnsson, J. I., & Bohlin, T. (2002). Can laboratory studies on dominance predict fitness of young brown trout in the wild? Behavioral Ecology and Sociobiology, 52(2), 102–108. 10.1007/s00265-002-0493-z

[ece37964-bib-0043] Höjesjö, J., Johnsson, J., & Bohlin, T. (2004). Habitat complexity reduces the growth of aggressive and dominant brown trout (*Salmo trutta*) relative to subordinates. Behavioral Ecology and Sociobiology, 56, 286–289. 10.1007/s00265-004-0784-7

[ece37964-bib-0044] Jaddot, C., Donnay, A., Ylieff, M., & Poncin, P. (2005). Impact implantation of a transmitter on *Sarpa salpa* behaviour: Study with a computerized video tracking system. Journal of Fish Biology, 67(2), 589–595. 10.1111/j.0022-1112.2005.00761.x

[ece37964-bib-0045] Johnsson, J. I., Jönsson, E., & Björnsson, B. T. (1996). Dominance, nutritional state, and growth hormone levels in rainbow trout (*Oncorhynchus mykiss*). Hormones and Behavior, 30(1), 13–21. 10.1006/hbeh.1996.0003 8724174

[ece37964-bib-0046] Jones, J., Börger, L., Tummers, J., Jones, P., Lucas, M., Kerr, J., Kemp, P., Bizzi, S., Consuegra, S., Marcello, L., Vowles, A., Belletti, B., Verspoor, E., Van de Bund, W., Gough, P., & Garcia de Leaniz, C. (2019). A comprehensive assessment of stream fragmentation in Great Britain. Science of the Total Environment, 673, 756–762. 10.1016/j.scitotenv.2019.04.125 31003103

[ece37964-bib-0047] Jones, P. E., Champneys, T., Vevers, J., Börger, L., Svendsen, J. C., Consuegra, S., Jones, J., & Garcia de Leaniz, C. (2021). Selective effects of small barriers on river‐resident fish. Journal of Applied Ecology, 58(7), 1487–1498. 10.1111/1365-2664.13875

[ece37964-bib-0048] Jungwirth, M., Schmutz, S., & Weiss, S. (1998). Fish migration and fish bypasses. Fishing News.

[ece37964-bib-0049] Kopack, C. J., Dale Broder, E., Lepak, J. M., Fetherman, E. R., & Angeloni, L. M. (2015). Behavioral responses of a highly domesticated, predator naïve rainbow trout to chemical cues of predation. Fisheries Research, 169, 1–7. 10.1016/j.fishres.2015.04.005

[ece37964-bib-0050] Kowalski, G. J., Grimm, V., Herde, A., Guenther, A., & Eccard, J. A. (2019). Does animal personality affect movement in habitat corridors? Experiments with common voles (*Microtus arvalis*) using different corridor widths. Animals, 9(6), 1–17. 10.3390/ani9060291 PMC6616401 31146468

[ece37964-bib-0051] Landsman, S. J., Wilson, A. D. M., Cooke, S. J., & van den Heuvel, M. R. (2017). Fishway passage success for migratory rainbow smelt *Osmerus mordax* is not dictated by behavioural type. River Research and Applications, 33(8), 1257–1267. 10.1002/rra.3176

[ece37964-bib-0052] Larsen, M. H., Thorn, A. N., Skov, C., & Aarestrup, K. (2013). Effects of passive integrated transponder tags on survival and growth of juvenile Atlantic salmon *Salmo salar* . Animal Biotelemetry, 1, 1–19. 10.1186/2050-3385-1-19

[ece37964-bib-0053] Lessells, C. M., & Boag, P. T. (1987). Unrepeatable repeatabilities: A common mistake. The Auk, 104, 116–121. 10.2307/4087240

[ece37964-bib-0054] Lothian, A. J., Schwinn, M., Anton, A. H., Adams, C. E., Newton, M., Koed, A., & Lucas, M. C. (2020). Are we designing fishways for diversity? Potential selection on alternative phenotypes resulting from differential passage in brown trout. Journal of Environmental Management, 262, 110317. 10.1016/j.jenvman.2020.110317 32250800

[ece37964-bib-0055] Metcalfe, N. B., Van Leeuwen, T. E., & Killen, S. S. (2016). Does individual variation in metabolic phenotype predict fish behaviour and performance? Journal of Fish Biology, 88, 298–321. 10.1111/jfb.12699 26577442 PMC4991269

[ece37964-bib-0056] Mirza, R. S., & Chivers, D. P. (2003). Response of juvenile rainbow trout to varying concentrations of chemical alarm cue: Response thresholds and survival during encounters with predators. Canadian Journal of Zoology, 81(1), 88–95. 10.1139/z02-216

[ece37964-bib-0057] Montiglio, P. O., Garant, D., Thomas, D., & Réale, D. (2010). Individual variation in temporal activity patterns in open‐field tests. Animal Behaviour, 80(5), 905–912. 10.1016/j.anbehav.2010.08.014

[ece37964-bib-0058] Noonan, M. J., Grant, J. W. A., & Jackson, C. D. (2012). A quantitative assessment of fish passage efficiency. Fish and Fisheries, 13(4), 450–464. 10.1111/j.1467-2979.2011.00445.x

[ece37964-bib-0059] Norman, G. R., & Streiner, D. L. (1994). Biostatistics: The bare essentials. Mosby.

[ece37964-bib-0060] Quinn, G. P., & Keough, M. J. (2002). Experimental design and data analysis for biologists. Cambridge University Press.

[ece37964-bib-0061] R Core Team (2014). R: A language and environment for statistical computing. R Foundation for Statistical Computing.

[ece37964-bib-0062] Richards, S. A. (2008). Dealing with overdispersed count data in applied ecology. Journal of Applied Ecology, 45, 218–227. 10.1111/j.1365-2664.2007.01377.x

[ece37964-bib-0063] Saunders, D. A., Hobbs, R. J., & Margules, C. R. (1991). Biological consequences of ecosystem fragmentation: A review. Conservation Biology, 5(1), 18–32. 10.1016/0006-3207(92)90725-3

[ece37964-bib-0064] Schwinn, M., Baktoft, H., Aarestrup, K., Lucas, M. C., & Koed, A. (2018). Telemetry observations of predation and migration behavior of brown trout (*Salmo trutta*) smolts negotiating an artificial lake. River Research and Applications, 34, 898–906. 10.1002/rra.3327

[ece37964-bib-0065] Sibly, R. M., Nott, H. M. R., & Fletcher, D. J. (1990). Splitting behaviour into bouts. Animal Behaviour, 39, 63–69. 10.1006/anbe.1993.1201

[ece37964-bib-0066] Sih, A., & Bell, A. M. (2008). Insight for behavioral ecology from behavioral syndromes. Advances in the Study of Behavior, 38, 227–281. 10.1016/S0065-3454(08)00005-3.Insights 24991063 PMC4075144

[ece37964-bib-0067] Sih, A., Bell, A., & Johnson, J. C. (2004). Behavioral syndromes: An ecological and evolutionary overview. Trends in Ecology and Evolution, 19(7), 372–378. 10.1016/j.tree.2004.04.009 16701288

[ece37964-bib-0068] Sih, A., Bell, A., Johnson, J. C., & Ziemba, R. (2004). Behavioural syndromes: An integrative overview. The Quarterly Review of Biology, 79(3), 241–277. 10.1086/422893 15529965

[ece37964-bib-0069] Sih, A., Cote, J., Evans, M., Fogarty, S., & Pruitt, J. (2012). Ecological implications of behavioural syndromes. Ecology Letters, 15(3), 278–289. 10.1111/j.1461-0248.2011.01731.x 22239107

[ece37964-bib-0070] Sih, A., Ferrari, M. C. O., & Harris, D. J. (2011). Evolution and behavioural responses to human‐induced rapid environmental change. Evolutionary Applications, 4, 367–387. 10.1111/j.1752-4571.2010.00166.x 25567979 PMC3352552

[ece37964-bib-0071] Silva, A. T., Lucas, M. C., Castro‐Santos, T., Katopodis, C., Baumgartner, L. J., Thiem, J. D., Aarestrup, K., Pompeu, P. S., O’Brien, G. C., Braun, D. C., Burnett, N. J., Zhu, D. Z., Fjeldstad, H. P., Forseth, T., Rajaratnam, N., Williams, J. G., & Cooke, S. J. (2018). The future of fish passage science, engineering, and practice. Fish and Fisheries, 19, 340–362. 10.1111/faf.12258

[ece37964-bib-0072] Smith, G. R., & Doupnik, B. L. (2005). Habitat use and activity level of large American bullfrog tadpoles: Choices and repeatability. Amphibia‐Reptilia, 26, 549–552. 10.1163/156853805774806197

[ece37964-bib-0073] Stamps, J., & Groothuis, T. G. G. (2010). The development of animal personality: Relevance, concepts and perspectives. Biological Reviews, 85(2), 301–325. 10.1111/j.1469-185X.2009.00103.x 19961473

[ece37964-bib-0074] Tentelier, C., & Piou, C. (2011). Obstacles to migration constrain nest distribution of Atlantic salmon. Ecology of Freshwater Fish, 20, 400–408. 10.1111/j.1600-0633.2010.00452.x

[ece37964-bib-0075] Thorlacius, M., Hellström, G., & Brodin, T. (2015). Behavioral dependent dispersal in the invasive round goby *Neogobius melanostomus* depends on population age. Current Zoology, 61(3), 529–542. 10.1093/czoolo/61.3.529

[ece37964-bib-0076] Thorstad, E. B., Økland, F., Aarestrup, K., & Heggberget, T. G. (2008). Factors affecting the within‐river spawning migration of Atlantic salmon, with emphasis on human impacts. Reviews in Fish Biology and Fisheries, 18(4), 345–371. 10.1007/s11160-007-9076-4

[ece37964-bib-0077] Tummers, J. S. (2016). Evaluating the effectiveness of restoring longitudinal connectivity for fish migration and dispersal in impacted river systems. University of Durham.

[ece37964-bib-0078] Tummers, J. S., Hudson, S., & Lucas, M. C. (2016). Evaluating the effectiveness of restoring longitudinal connectivity for stream fish communities: Towards a more holistic approach. Science of the Total Environment, 569–570, 850–860. 10.1016/j.scitotenv.2016.06.207 27423105

[ece37964-bib-0079] van Leeuwen, T. E., Hughes, M. R., Dodd, J. A., Adams, C. E., & Metcalfe, N. B. (2016). Resource availability and life‐history origin affect competitive behavior in territorial disputes. Behavioral Ecology, 27, 385–392. 10.1093/beheco/arv163

[ece37964-bib-0080] van Leeuwen, T. E., Rosenfeld, J. S., & Richards, J. G. (2010). Failure of physiological metrics to predict dominance of juvenile Pacific salmon (*Oncorhynchus* spp.): Habitat effects on the allometry of growth in dominance hierarchies. Canadian Journal of Fisheries and Aquatic Sciences, 68, 1811–1818.

[ece37964-bib-0081] Vollset, K. W., Lennox, R. J., Thorstad, E. B., Auer, S., Bär, K., Larsen, M. H., Mahlum, S., Näslund, J., Stryhn, H., & Dohoo, I. (2020). Systematic review and meta‐analysis of PIT tagging effects on moratlity and growth of juvenile salmonids. Reviews in Fish Biology and Fisheries, 30, 553–568. 10.1007/s11160-020-09611-1

[ece37964-bib-0082] Wilson, A. D. M., Hayden, T. A., Vandergoot, C. S., Kraus, R. T., Dettmers, J. M., Cooke, S. J., & Krueger, C. C. (2017). Do intracoelomic telemetry transmitters alter the post‐release behaviour of migratory fish? Ecology of Freshwater Fish, 26(2), 292–300. 10.1111/eff.12275

[ece37964-bib-0083] Wilson, D. S., Coleman, K., Clark, A. B., & Biederman, L. (1993). Shy‐bold continuum in pumpkinseed sunfish (*Lepomis gibbosus*): An ecological study of a psychological trait. Journal of Comparative Psychology, 107, 250–260. 10.1037/0735-7036.107.3.250

[ece37964-bib-0084] Winter, E. R., Tummers, J. S., Aarestrup, K., Baktoft, H., & Lucas, M. C. (2016). Investigating the phenology of seaward migration of juvenile brown trout (*Salmo trutta*) in two European populations. Hydrobiologia, 775(1), 139–151. 10.1007/s10750-016-2720-z

[ece37964-bib-0085] Woltz, H. W., Gibbs, J. P., & Ducey, P. K. (2008). Road crossing structures for amphibians and reptiles: Informing design through behavioral analysis. Biological Conservation, 141(11), 2745–2750. 10.1016/j.biocon.2008.08.010

